# Conundrum of station 13 lymph nodes in Gallbladder Carcinoma: Retrospective tryst with a forgotten entity

**DOI:** 10.1007/s13304-025-02237-7

**Published:** 2025-05-13

**Authors:** Mayank Tripathi, Kumar Vineet, Nitesh Joshi, Ankita Pal, Tejas Vispute, Akhil Kapoor, Paramita Paul

**Affiliations:** 1 Department of Surgical Oncology, MPMMCC & HBCH, Tata Memorial Centre, Homi Bhabha National Institute, Varanasi, India; 2 Clinical Research Secretariat, MPMMCC & HBCH, Tata Memorial Centre, Homi Bhabha National Institute, Varanasi, India; 3 Department of Medical Oncology, MPMMCC & HBCH, Tata Memorial Centre, Homi Bhabha National Institute, Varanasi, India; 4 Department of Onco Pathology, MPMMCC & HBCH, Tata Memorial Centre, Homi Bhabha National Institute, Varanasi, India

**Keywords:** Gallbladder carcinoma, Station 13, Disease-free survival, Stage IIIB, Stage IVB

## Abstract

Gallbladder cancers are one of the lethal cancers with dismal prognosis. There is discrepancy between various biliary societies regarding extent of regional lymph nodes of gallbladder. Some consider station 13 as regional nodes, whereas others consider it as distant metastatic node. This is a retrospective analysis of disease-free survival of stage IIIB and IVB-M0 patients in gall bladder cancer with positive station 13 lymph node. Electronic medical records of patients were used to retrieve the data. The mean and median disease-free survival of gallbladder carcinoma patients with station 13 lymph-node positivity is 16.2 months (95% CI 11.57–20.85 months) and 12 months (95% CI 9.57–14.43 months), respectively. Disease-free survival of Stage IIIB and IVB-M0 gallbladder carcinoma patients with station 13 lymph-node positivity is better than overall survival of stage IVB-M1 patients and supports the inclusion of station 13 lymph node as regional lymph-nodal basin of gall bladder carcinoma. This study paves the way for a large prospective study to consider station 13 lymph node as regional lymph node in gallbladder cancer.

## Introduction

Gallbladder carcinoma (GC) ranks as the 25 th most frequent cancer globally according to the GLOBOCAN 2020 statistics. It exhibits a higher incidence in females than males [1]. India has substantial burden of GC. Early stage GC have 5-year survival rate in the range of 30–40% and locally advanced lesions have dismal 1-year survival rate of around 10% [2]. The 8 th edition of the American Joint Committee on Cancer (AJCC) staging manual classifies nodal involvement in GC as N1 or N2 depending on the number of positive lymph nodes in the regional lymph-nodal basin. N1 indicates metastasis to 1–3 regional lymph nodes, while N2 refers to metastasis to 4 or more regional lymph nodes [3]. According to the AJCC, regional lymph nodes include those along the common bile duct, hepatic artery, portal vein, and cystic duct. However, the classification does not clarify whether station 13 lymph nodes—located posterior to the head of the pancreas—should be considered regional or distant metastatic nodes [4]. The National Comprehensive Cancer Network (NCCN) guidelines, aligned with the AJCC, similarly define regional lymph nodes in GC as those within the hepatic portal area.

Conversely, the Japanese Society of Hepato-Biliary-Pancreatic Surgery (JSBS) and the Chinese Journal of Surgery guidelines classify station 13a lymph nodes as regional for GC, recommending their dissection along with station 8 and station 12 lymph nodes during surgery [5]. Historically, the 7 th edition of the AJCC staging manual defined N1 as metastasis to nodes along the cystic duct, hepatic artery, common bile duct and portal vein; and N2 as metastasis to retroperitoneal lymph nodes, such as peri-aortic, pericaval, superior mesenteric artery, and/or celiac artery lymph nodes. However, the status of nodes located on the posterior aspect of the head of the pancreas (station 13) remained ambiguous.

Our center located in a high-incidence region for GC treats station 13a and 13b lymph nodes as regional lymph nodes, routinely dissecting them during radical or revision cholecystectomy.

This study aims to assess the prognostic impact of positivity of station 13 lymph nodes in GC.

## Materials and methods

This is a retrospective exploration of prospectively maintained data from a high-volume cancer center. This study included all consecutive patients over 18 years of age who underwent surgery for GC at our institute between May 1, 2018, and December 31, 2023. Data were retrieved from electronic medical records; and a waiver of consent was obtained, given that all information was collected retrospectively. Ethical clearance for the study was approved by the institutional ethics committee. Declaration of Helsinki was followed throughout study.

The primary objective was to determine disease-free survival (DFS) in patients who underwent surgery for GC and had positive station 13 lymph nodes identified in the final histopathology report.

Radical cholecystectomy was performed as surgery for treatment naïve GC and revision radical cholecystectomy was performed for incidental GC diagnosed after simple cholecystectomy. Surgical template at our institute includes removal of gallbladder along with liver wedge taking 2 cm margin of liver, removal of lymph-node station 8 a; 12 a, b, p; 13 a, b and 16 b1. If 16 b1 lymph node comes out to be positive on final histopathology, then it is considered as distant metastasis. Each lymph-node station was removed separately.

According to the extent of the tumor (T), extent of spread to the lymph nodes (N), and presence of metastasis (M) in GC of AJCC 8 th edition, none of our patients in this study had T1 or T4 disease. Patients with positive station 16 b1 lymph nodes were excluded from study. Patients with 1–3 positive lymph nodes were considered as stage IIIB and those with 4 or more than 4 positive lymph nodes were considered as stage IVB-M0.

DFS was measured from the date of surgery to the date of recurrence, as determined by follow-up imaging. For patients who died due to causes unrelated to cancer (COVID-19 and sepsis), the date of death was considered the last day of DFS. For those who defaulted on follow-up, the last recorded follow-up date was used as the DFS endpoint. For patients still under follow-up without signs of recurrence, their data were censored as on July 31, 2024.

Kaplan–Meier analysis was used to estimate survival rates for a disease-free state.

All N-positive patients received adjuvant chemotherapy in the form of six cycles of cisplatin and gemcitabine or oxaliplatin and gemcitabine.

None of the patients received adjuvant radiation therapy or neoadjuvant chemotherapy/radiation therapy.

## Results

**Fig. 1:**
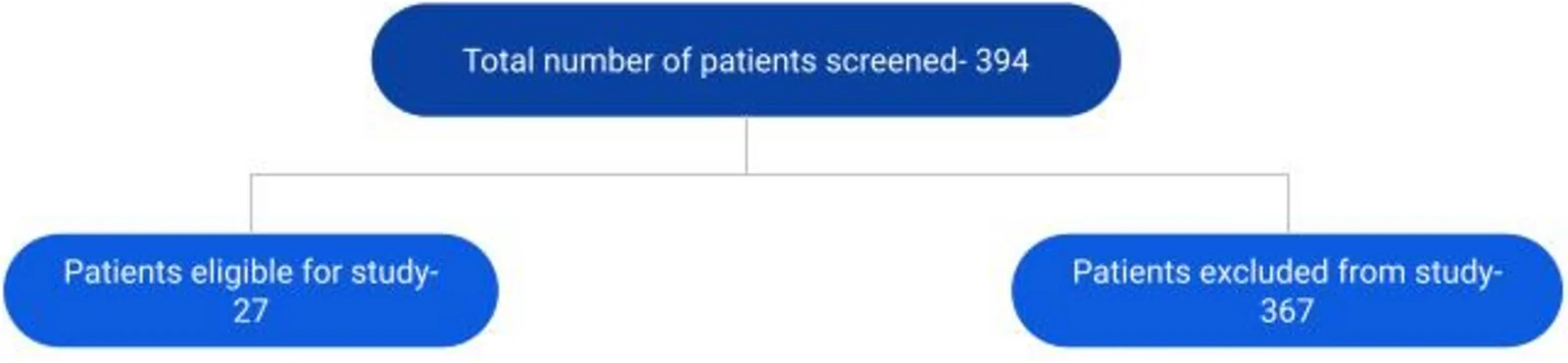
Flow diagram of the study population

A total of 394 consecutive patients who underwent surgery for GC during the study period were screened, out of which 27 were eligible for analysis. Therefore, this study included 27 patients with positive station 13 lymph nodes (Fig. 1). Clinicopathological characteristics of the study population are presented in Table 1. The median follow-up duration of the cohort was 25 months [inter-quartile range (IQR) 12–31 months]. 20 patients experienced disease recurrence, while 7 patients were censored, that is, there were no signs of recurrence on follow-up at the time of analysis. The percentage of censored patients was 25.9%.

**Table 1:** Clinicopathological characteristics of the patients'

Patients’ characteristics	Values
Mean age	53.8 years
Median age	55 years
Number of males	4
Number of females	23
Percentage of incidental GC	25.9% i.e. 7 cases
Percentage of minimally invasive approach	0
Postoperative mortality	0
Postoperative major morbidity—Classified as Clavien–Dindo Grade 3 and above	0
Percentage of T2 cancers	44.4% to 12 cases
Percentage of T3 cancers	55.6% to 15 cases
Number of R1 resection	0
Percentage grading of tumors	Grade 1- 14.8% Grade 2- 59.3% Grade 3- 25.9%
Mean of harvested lymph nodes	16.5
Median of harvested lymph nodes	15
Median number of positive lymph nodes in N1	1
Median number of positive lymph nodes in N2	4
Percent of local recurrence	0
Percent of distant recurrence- in patients' who had recurrence	100%

**Fig. 2:**
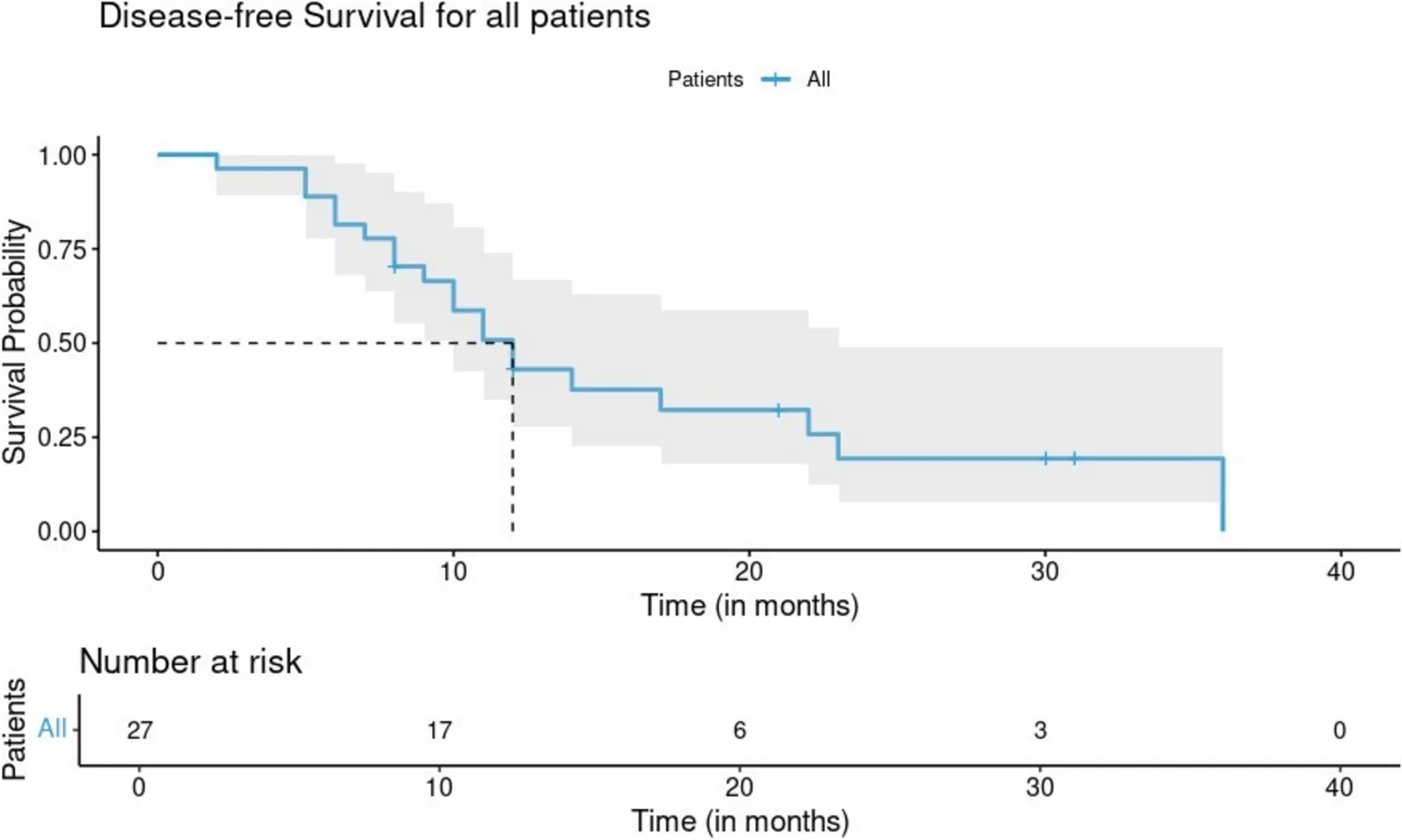
Disease-free survival curve obtained from the Kaplan–Meier method shows the survival of the cohort

The mean disease-free survival time was estimated at 16.2 months (95% CI 11.57–20.85 months). The median disease-free survival time was 12 months (95% CI 9.57–14.43 months). The 1-year disease-free survival rate (recurrence-free rate) of the cohort was 50.8 ± 9.8% (Table 2 and Fig. 2).

**Table 2:** DFS by Kaplan–Meier method of all patients

Disease-free survival							
Mean survival time				Median survival time			
Estimate	Std. Error	95% Confidence interval		Estimate	Std. error	95% Confidence interval	
		Lower Bound	Upper Bound			Lower Bound	Upper Bound
16.209	2.367	11.569	20.849	12.000	1.241	9.567	14.433

16 patients were in stage IIIB and 11 patients were in stage IVB-M0. In the stage IIIB group, 12 patients experienced a disease recurrence, while 4 patients were censored resulting in 25% of the stage IIIB being censored. In contrast, 8 stage IVB-M0 patients experienced events, and 3 were censored (27.3%). Therefore, there was not much difference between the two stages which shows that around a quarter of the patients in each group were still disease free at the end of the study.

**Fig. 3:**
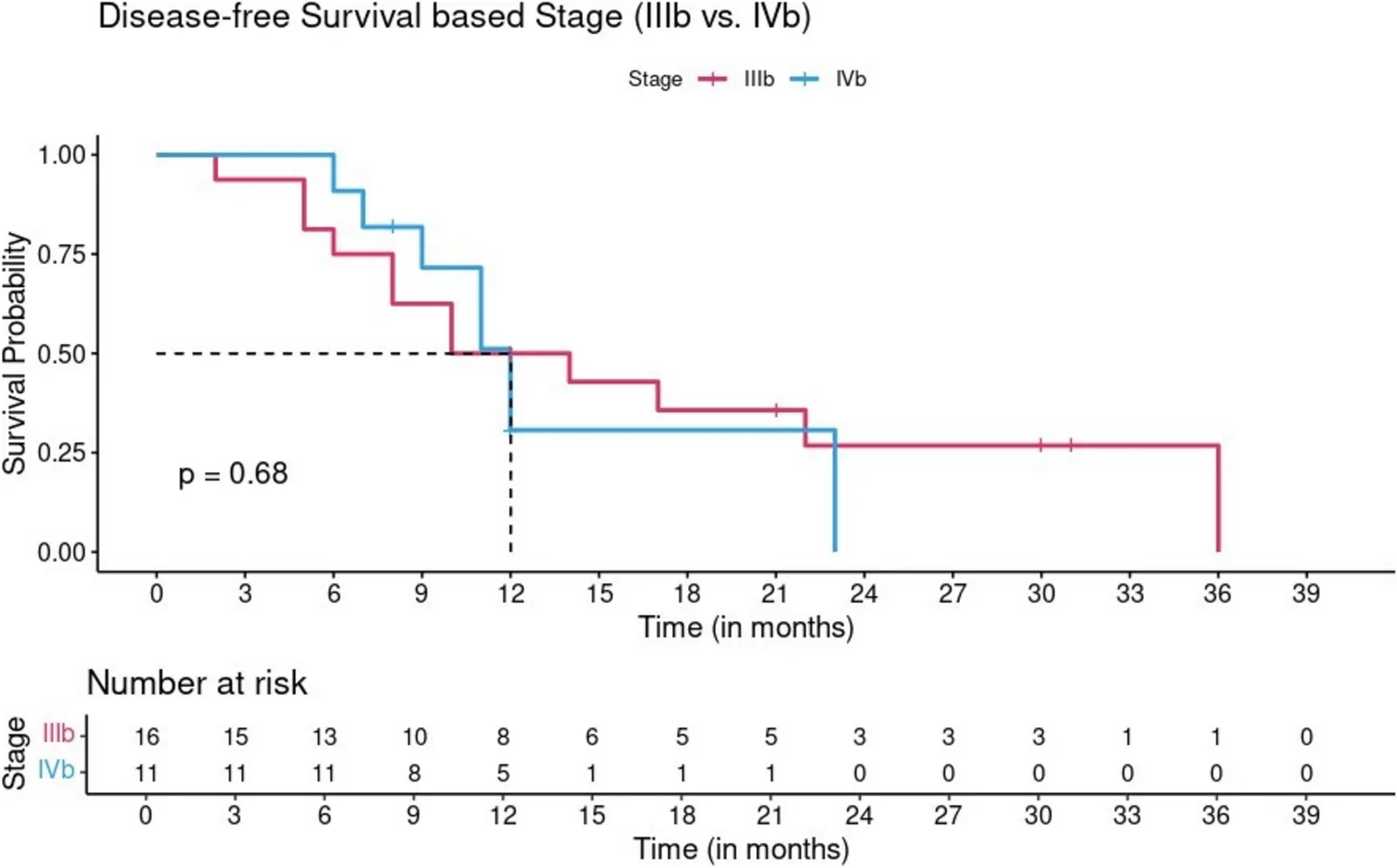
Disease-free survival curve obtained from the Kaplan–Meier method shows the survival of the cohort based on Stages (IIIB vs IVB-M0)

For patients with Stage IIIB disease, the mean disease-free survival time was estimated at 17.2 months (95% CI 10.62–23.78 months). The median disease-free survival time for this group was 10 months (95% CI 2.52–17.48 months). This indicates that half of the Stage IIIB patients experienced disease recurrence within 10 months. For patients with Stage IVB-M0 disease, the mean disease-free survival time was 13.9 months (95% CI 9.68–18.05 months). The median disease-free survival time for Stage IVB-M0 patients was 12 months (95% CI 9.89–14.11 months). The *p* value from the log-rank test was 0.68, indicating no statistically significant difference in disease-free survival between patients with Stage IIIB and Stage IVB-M0 GC (Table 3 and Fig. 3).

**Table 3:** DFS by Kaplan–Meier method of patients based on Stage

Disease-free survival								
Stage	Mean survival time				Median survival time			
	Estimate	Std. error	95% Confidence interval		Estimate	Std. error	95% Confidence interval	
			Lower bound	Upper bound			Lower bound	Upper bound
IIIB	17.196	3.357	10.617	23.776	10.000	3.818	2.516	17.484
IVB-M0	13.864	2.134	9.680	18.047	12.000	1.076	9.892	14.108

These results suggest that while Stage IIIB patients had a slightly higher mean survival time, the difference in survival between the two stages was not statistically significant.

Notably, 9 out of 16 N1 patients had isolated positivity in station 13 lymph nodes, with no involvement of station 8 or 12 nodes.

## Discussion

In the 1990's, Japanese researchers mapped the lymphatic spread pathways of GC. Uesaka et al. described three main routes of lymphatic metastasis. The “right route”, the most common, involves drainage from common bile duct lymph nodes to station 13a or periportal nodes, eventually reaching peri-aortic lymph nodes. In the"left route,"drainage starts at the gallbladder triangle lymph nodes, then proceeds to station 13 lymph nodes before passing through hepatoduodenal ligament nodes and ultimately reaching peri-aortic nodes. The “portal route” involves direct drainage from hilar nodes to peri-aortic nodes [6]. Ito et al. and Shirai et al. also tried to devise the lymphatic spread of biliary tract cancers. Ito mapped three pathways, cholecysto-retropancreatic pathway, cholecysto-celiac pathway, and cholecysto-mesenteric pathway [7, 8].

The JSBS's 3rd Biliary Tract Cancers Classification considers several lymph-node stations, including 12 a, b, p, c, 8 a, p, and 13a, as regional for GC, classifying them as N1. Rest other lymph nodes are classified as non-regional distant metastatic lymph nodes [9]. Our findings, with 9 patients showing isolated station 13 positivity, suggest a direct lymphatic pathway from the gallbladder to these nodes, challenging the current AJCC classification. In another study, isolated positivity of station 13 lymph node was found in 3 out of 27 patients with single positive node in GC [10].

Sakata et al. reported a 5-year overall survival (OS) rate of 31.6% for patients with positive station 13a lymph nodes, concluding that their incidence and survival impact were similar to other regional nodes, thus advocating for their inclusion as regional nodes [11]. An SEER analysis from 2023 reported 5-year survival rates of 24.1% and 10% for stage IIIB and IVB patients, respectively [12]. Another SEER study identified a median OS of 19 months for GC patients [13]. A study from hotbed of GC showed the combined 1-year OS of Stage IIIB and IVB patients as 11.8% [14]. All these data show the dismal prognosis of node positive non-metastatic GC.

Another interesting point in our cohort was site of recurrence. None of our patients recurred locoregionally and all of them had distant recurrence. In a study of 274 GC patients, more than 65% of patients had distant recurrence [15]. Similar recurrence pattern was observed in the study by Peng et al. and Margonis et al. [16, 17]. Yuan et al. noted in post-operative GC that station 13 is a common site of recurrence among the potential target volume of post-operative radiotherapy [18]. This is an intriguing manifestation; it shows our template of radical cholecystectomy is adequate, since none of the patient had locoregional recurrence.

Our study is the first, to our knowledge, to focus specifically on DFS for GC patients with positive station 13 lymph nodes. The mean DFS is 16.2 months, and the 1-year DFS is around 50%, suggesting that station 13 lymph-node positivity is not equivalent to distant metastatic disease (stage IVB-M1). This supports the view, particularly from the Japanese studies, that station 13a nodes should be resected and classified as regional.

At our center, both station 13a and 13b nodes are treated as regional and are resected en-bloc.

The limitation of our study is its small sample size and retrospective nature. The DFS for GC patients with positive station 13 lymph nodes in our study needs to be validated in a large prospective study. Despite its limitations, this study is important in view that station 13 in GC, which can be easily resected while sampling of inter-aortocaval lymph nodes, does not have a consensus over its status as regional or metastatic lymph node. Furthermore, in our cohort, many cases only have positive station 13 lymph nodes. Our study raises the legitimate argument of clarifying the position of this forgotten entity in GC staging.

## Conclusion

Since many biliary societies are already considering station 13 lymph node as regional lymph node, now it is a ripe time for future revisions of the AJCC classification to align with evidence emerging from high-incidence regions and clear up the conundrum surrounding station 13 lymph nodes in GC.

## Data Availability

Data can be provided on reasonable request.
